# Evaluation of Risk Perception of Smoking after the Implementation of California’s Tobacco 21 Law

**DOI:** 10.3390/ijerph192416971

**Published:** 2022-12-17

**Authors:** Joanna K. Sax, Neal Doran

**Affiliations:** 1 California Western School of Law, 225 Cedar St., San Diego, CA 92101, USA; 2 Department of Psychiatry, University of California San Diego, 9500 Gilman Drive, La Jolla, CA 92093, USA

**Keywords:** Tobacco 21 law, smoking, risk perception, intervention, public policy, young adults

## Abstract

Decreasing smoking initiation remains a public health priority. In 2016, California, in the United States, enacted the Tobacco 21 law, which raised the minimum age for the purchase of tobacco products from age 18 to age 21. This paper evaluates whether the enactment and implementation of the Tobacco 21 law changed how young adults perceive the risk(s) of smoking. Data were drawn from a cohort of emerging adults (*n* = 575) in California who were non-daily smokers at enrollment and followed quarterly for 3 years. Data were collected during 2015–2019. Piecewise multilevel regression models were used to test for changes in smoking status and perceived risks of cigarettes after Tobacco 21 enforcement began. Findings indicated that the prevalence of current smoking and perceived risks of smoking both declined following Tobacco 21 implementation (*p*s < 0.001). Post-hoc analyses suggested that post-implementation changes in perceived risk occurred primarily among ongoing smokers. Findings suggest that Tobacco 21 and associated public health measures have been effective, but additional research is needed to disentangle the effects of specific components. Understanding the impact and efficacy of tobacco laws provides great social value to research and implement policies that create intervention(s) on reducing tobacco use initiation.

## 1. Introduction

In 2016, California, in the United States, enacted Senate Bill No. 7 (known as “Tobacco 21”), which raised the minimum age for sales of tobacco products from age 18 to 21 [1]. The purpose of this Tobacco 21 law was to make it more difficult for individuals under the age of 21 to obtain cigarettes and other tobacco and nicotine-containing products [2,3]. Previous research indicates that individuals are more likely to become addicted to smoking the earlier they begin [2]. Historically, the initiation of smoking primarily occurred during adolescence, but recent data suggest that initiation is shifting to young adulthood [4]. Thus, the Tobacco 21 law raised the minimum age for retailers to sell nicotine and tobacco products to individuals. In conjunction with the minimum age increase, California launched a public awareness campaign to educate the public, support the minimum age change in the Tobacco 21 law, and inform retailers about the change [5].

Previous studies indicate that Tobacco 21 laws can be an effective means of reducing smoking prevalence, particularly among youth and emerging adults [6,7]. Evidence indicates that such restrictions decrease the ability of individuals under 21 to purchase tobacco products, potentially delaying or even preventing the initiation of tobacco use [8]. It has been demonstrated that California retailers were highly aware of the law and generally compliant with its requirements [5].

While the Tobacco 21 law appears to decrease access to tobacco products at the point of sale for individuals under 21, it is less clear whether it impacted how young adults perceive the risk(s) of smoking. This is an important question, given that the perceived risk of smoking is inversely associated with the likelihood of initiation [9,10]. That is, if Tobacco 21 laws increase the perceived risk in addition to reducing tobacco access for 18–20-year-olds, they may be more likely to prevent rather than merely delay initiation. To our knowledge, the present study is the first to examine changes in perceived risks from smoking among young adults following the implementation of the Tobacco 21 law.

The primary goal of this study was to test two hypotheses in a sample of young adults who had been non-daily smokers at enrollment. First, we expected that the likelihood of current cigarette smoking would decline following Tobacco 21 implementation. Second, we predicted that perceived risks of smoking would increase following the implementation of Tobacco 21. Secondary analyses examined associations with smoking status.

## 2. Materials and Methods

### 2.1. Participants

Participants (*n* = 575) were young adults enrolled in a longitudinal study of non-daily cigarette smoking. Eligibility requirements included being aged 18–24 at baseline, smoking cigarettes at least monthly for the past 6 months or more, never having been a daily smoker, and being a California resident at the time of enrollment. A total of 52% of percent of participants identified as male, 44% as non-Hispanic White, 18% as Asian American, and 22% as Hispanic/Latinx. The average age at baseline was 20.4 years (SD = 1.7).

### 2.2. Procedure

Candidates were recruited via paid Facebook posts. If interested, they completed a brief online eligibility screener. Eligible respondents completed a baseline assessment and then quarterly assessments for the next 3 years, yielding a total of 13 waves of assessment. All assessments occurred online. At annual assessment points (baseline and 1, 2, and 3 years later; hereafter annual waves), all measures were completed on a single day, and participants were compensated with $25 gift cards. At each of the 9 waves between these annual assessments (hereafter quarterly waves), participants completed brief daily assessments for 9 consecutive days and were compensated up to $40 per wave. Data were collected during 2015–2019. The study was approved by the Institutional Review Board at the University of California, San Diego.

### 2.3. Measures

*Demographics* evaluated at baseline included self-identified sex, racial/ethnic identity, and age. Due to small cell sizes for some groups, race/ethnicity was collapsed into 4 groups: non-Hispanic White (*n* = 254), Asian American (*n* = 102), Latinx (*n* = 122), and multiple or other backgrounds (*n* = 96).

*Cigarette use* was assessed at each wave. At annual waves, a Timeline Followback procedure [11] was used to evaluate the number of cigarettes smoked in each of the past 14 days. At quarterly waves, participants reported the number of cigarettes smoked in the past 24 h on each of 9 consecutive days. From these raw data, we calculated a *cigarette days* variable, which reflected the number of days on which cigarettes were smoked at each wave, and a binary *smoking status* variable, indicating whether participants had smoked at each wave (coded as 0 = no cigarette days, 1 = 1 or more cigarette days). Because the maximum number of smoking days differed for annual and quarterly assessments, we also calculated *days assessed*, reflecting the number of days at each wave that smoking was assessed, for use as a covariate.

*Risk perceptions* were measured using the Negative Consequences subscale from the short form of the Smoking Consequences Questionnaire [12,13], which has been validated for use in young adult samples. The subscale includes 4 items on which participants rate perceived health risks from cigarette smoking on a 10-point scale. The subscale had good internal consistency in the present sample (α = 0.85). Risk perceptions were evaluated at each annual wave and on day 9 of each quarterly wave.

### 2.4. Analytic Plan

Two primary models were fit. First, a longitudinal logistic regression model using the generalized estimating equations (GEE) approach [14] was used to evaluate post-implementation change in smoking status. Second, a mixed effects longitudinal regression model was used to evaluate post-implementation change in the perceived risk of smoking. In light of previous reports of smoking differences between demographic groups, racial/ethnic background and gender were included as covariates in hypothesis tests [15]. Similarly, we included total cigarettes smoked in the past 2 weeks at baseline as a covariate. Our primary interest was in the extent to which smoking status and the perceived risk from cigarette smoking changed over time and in relation to the implementation of Tobacco 21 in California.

Both models utilized a piecewise or segmented multilevel longitudinal regression approach, which has been recommended [16] for the evaluation of policy changes. This piecewise approach included segments for the periods before and after Tobacco 21 implementation, allowing us to compare outcomes between the two periods, as well as change over time following implementation. Models included a binary, time-varying *Tobacco 21* variable that indicated whether each measurement of perceived risk occurred before or after implementation in June 2016. We also created a *post-implementation slope* variable to evaluate change over time after implementation; for each participant, *post-implementation slope* was coded as 0 for all visits prior to June 2016, 1 for the first visit after implementation, 2 for the second, and so on. Finally, we created a binary, time-varying *age21* variable that reflected whether participants were 21 years old at the time of their participation in each wave. All analyses were conducted using Stata 15.0 (StataCorp LLP, College Station, TX, USA) with alpha = 0.05. Analyses utilized an intent-to-treat approach, retaining participants who were lost to follow-up without imputing missing values [17].

## 3. Results

### 3.1. Preliminary Analyses

Participants completed a total of 6032 assessments of cigarette use and risk perceptions; 74% of these occurred after Tobacco 21 implementation. At the participant level, 90% had at least one assessment prior to implementation, 79% had at least two, 57% had at least three, 37% had at least four, and 8% had five. In other words, for almost all participants, the key variables were evaluated at least once prior to implementation, and the majority of participants reported cigarette use and perceived risk at three or more waves prior to implementation. Attrition increased gradually over the 3-year study period, from 2% at the first baseline wave to 11% at year 1, 23% at year 2, and 37% at year 3 regarding missing smoking status and/or risk perception data. Baseline demographic and cigarette use characteristics are shown in Table 1.

### 3.2. Hypothesis Tests: Post-Implementation Changes

The piecewise GEE model of smoking status is shown in Table 2. The *time* term was not significant, indicating that smoking status was stable prior to implementation. However, the *tobacco 21* (*z* = −2.16, *p* = 0.031) and *post-implementation slope* (*z* = −3.20, *p* = 0.001) were both significant, and both indicated an inverse association with smoking status. That is, current smoking was less likely after the implementation of Tobacco 21 and became increasingly unlikely during the post-implementation period. Smoking status was not associated with sex, race/ethnicity, or whether participants were 21 or older.

The piecewise mixed regression model of risk perceptions is shown in Table 3. Time was inversely associated with perceived risk from smoking (*z* = −3.90, *p* < 0.001), indicating that perceived risk was declining during the period prior to Tobacco 21 implementation. However, both the tobacco 21 (*z* = 2.80, *p* = 0.005) and post-implementation slope (*z* = 3.95, *p* < 0.001) terms were positively associated with perceived risk. This indicates that participants reported greater perceived risk from cigarette smoking after Tobacco 21 implementation compared with before and that the strength of this association was increasing over time. Risk perceptions did not differ by race or whether participants were age 21 but were higher among participants who identified as female, who reported heavier cigarette use at baseline, and who smoked cigarettes more frequently.

**Figure 1:**
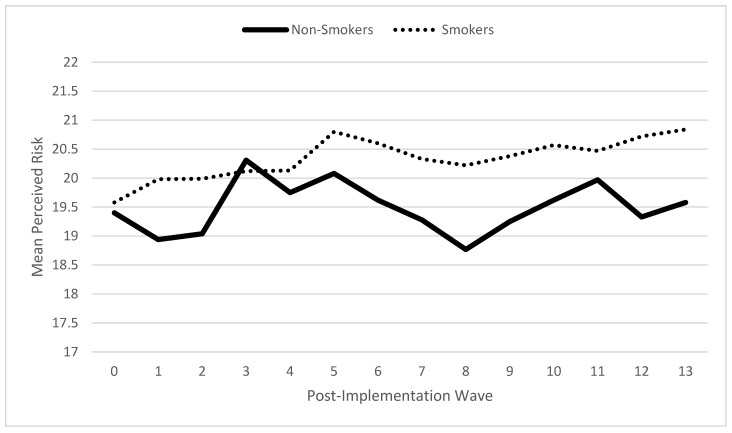
Risk perceptions over time by smoking status after Tobacco 21 implementation.

To better understand this finding, post hoc analyses examined change in perceived risk over time during the post-implementation period as a function of smoking status (i.e., whether participants reported any smoking days at each individual post-implementation wave; see Figure 1). These analyses excluded pre-implementation waves and utilized longitudinal mixed effects regression, with time-varying risk perceptions as the outcome variable. For non-smokers, the perceived risk did not change over time after implementation (*z* = −0.50, *p* = 0.615). In contrast, for those who reported continued smoking, perceived risks from smoking increased over the course of the post-implementation waves (*z* = 3.48, *p* = 0.001).

## 4. Discussion

This study investigated smoking status and perceived risk of cigarette smoking among young adults before and after the statewide implementation of Tobacco 21 in California, United States. As hypothesized, we found that likelihood of smoking decreased, and risk perception increased after the implementation of the Tobacco 21 law. We also found that the increased perception of risk was associated with smoking status, increasing among those who were current smokers but not those who were not.

At the time of enactment, California was among the minority of states in the United States that adopted a Tobacco 21 law. Other states and countries have a strong interest in preventing young adults from initiating tobacco use due to the individual health and public health issues related to smoking. Given the experience with state-initiated Tobacco 21 laws, the United States federal government enacted its own Tobacco 21 law [18]. Understanding not only the efficacy of a Tobacco 21 law but the possible underlying reason(s) for its efficacy provides great social value in engaging in effective legal interventions to address public health issues, both as it relates to tobacco use and possibly more broadly to address other public health issues.

Studies analyzing risk perception suggest that the more heavily regulated an industry is, the higher individuals perceive the risk of that industry [19]. Thus, one possible reason that the Tobacco 21 law was associated with an increased perception of risk is because it represented increased regulation of tobacco products. In addition, the public awareness campaign may also have contributed to perceptions of risk.

Additional studies are needed to increase our understanding of factors that directly impact perceived risk to enable the optimization of public health messaging. For example, risk information may be perceived differently depending on the perceived authority of the source (e.g., television news versus social media) [20]. Initial work examining message content also suggests that because the harms of combustible tobacco are well-understood, messages that focus on emotional content framed around loss may be more effective than messages that focus on providing information [21].

This study suggests that the impact of restrictions may go beyond logistical barriers by impacting attitudes toward products or activities that are regulated. This is important because it suggests that interventions that impact the perceived risks of tobacco products can contribute to alleviating the public health consequences of tobacco use. Clinical implications of decreasing the initiation and prevalence of smoking among young adults include a decrease in mortality and morbidity [2]. These clinical implications are not just for the individual but also for maternal/fetal and infant outcomes if tobacco use is lowered among young adults [2].

Legal interventions do not always obtain the desired result and/or may have trade-offs [22]. The results of this study suggest that the Tobacco 21 law may have led to increased risk perception among emerging adults who were using tobacco products. This is potentially important for multiple reasons. First, previous studies have indicated that greater perceived risk is associated with a lower likelihood of smoking uptake and a greater likelihood of cessation [9,10]. Second, evidence indicates that the perceived risk of smoking cigarettes has been declining in recent years [23], which raises concern that prevalence may increase. Our data suggest that Tobacco 21 laws may be an effective way to address this issue. In addition, these findings provide insight into the potential for legal policy interventions to align an individual’s perception of risk with the evidence-based assessment of risk.

To our knowledge, this is the first study to demonstrate that the Tobacco 21 law appears to change risk perception. Previous studies, for example, analyzed retailer compliance and point of sale to young adults, which focus on the efficacy of restricting access to young adults [5,8]. This study goes beyond compliance to suggest that this legal policy intervention changed attitudes toward tobacco use. Put differently, the Tobacco 21 law appears effective not just because it made it more difficult for young adults to purchase tobacco products but because it also changed underlying attitudes towards the risk of smoking.

This study has several limitations. First, we did not directly evaluate factors that may have impacted risk perceptions over time. It could be that the messaging in the public media campaign impacted risk perception. Alternatively, other components of Tobacco 21 implementation (e.g., restricted access at the point of sale) may have been significant. Second, all participants were young adults with a history of non-daily cigarette smoking who were California residents at the time of enrollment. It is possible that findings are not generalizable to other groups.

The impact of the Tobacco 21 law on the risk perception of young adults is important both as an intervention for reducing tobacco use initiation and could have wider application and insight in other areas in which aligning individual perception of risk with the evidence-based assessment of risk provides great social value, such as vaccines, food, and other public health issues.

## 5. Conclusions

Smoking among young adults continues despite several public health measures to curtail smoking initiation. These data suggest that the enactment of the Tobacco 21 law effectively and significantly changed the perception of risk among young adults, especially those who were current smokers. Despite this significant change, young adults continue to smoke; thus, future studies that aim to understand why risk perception changed may inform future interventions to increase the extent to which they effectively reduce cigarette consumption.

## Figures and Tables

**Table 1:** Demographic and clinical characteristics.

Variable	M (SD), Range or Proportion
Age at baseline	20.4 (1.7), 18–24
Aged 21+ at baseline	33.9%, *n* = 195
Aged 21+ at year 1	63.5%, *n* = 365
Aged 21+ at year 2	87.2%, *n* = 501
% identifying as female	51.7%, *n* = 297
% identifying as non-Hispanic White	48.7%, *n* = 280
Total cigarettes, past 14 days at baseline	12.4 (17.7), 0–209
Perceived risk of smoking at baseline	19.6 (5.8), 4–28

**Table 2:** Piecewise longitudinal GEE model of smoking status overall and after Tobacco 21 implementation.

Predictor	Coefficient	Std Err	*z*-Score	*p*-Value
Days assessed	0.05	0.01	11.91	<0.001
Gender ^a^	−0.03	0.03	−1.17	0.240
Racial/ethnic identity ^b^	0.02	0.02	0.99	0.323
Total cigarettes at baseline	0.01	0.01	3.67	<0.001
Time	−0.01	0.01	−0.93	0.352
Age 21 ^c^	0.04	0.03	1.23	0.217
Tobacco 21 ^d^	−0.08	0.04	−2.16	0.031
Post-implementation slope	−0.03	0.01	−3.20	0.001

Note: ^a^ 0 = male, 1 = female; ^b^ 0 = non-Hispanic White, 1 = Hispanic or Latino, 2 = Asian American, 3 = other or multiple backgrounds; ^c^ 0 = participant was aged 18–20 at the time of assessment, 1 = participant was aged 21 or older at the time of assessment; ^d^ 0 = assessment occurred prior to Tobacco 21 implementation; 1 = assessment occurred after Tobacco 21 implementation.

**Table 3:** Piecewise longitudinal multilevel model of risk perceptions overall and after Tobacco 21 implementation.

Predictor	Coefficient	Std Err	*z*-Score	*p*-Value
Days assessed	−0.11	0.03	−3.95	<0.001
Gender ^a^	0.88	0.15	5.67	<0.001
Racial/ethnic identity ^b^	0.02	0.09	0.27	0.789
Total cigarettes at baseline	0.04	0.01	8.02	<0.001
Time	−0.23	0.06	−3.90	<0.001
Age 21 ^c^	0.25	0.19	1.31	0.189
Cigarette days	0.16	0.03	6.10	<0.001
Tobacco 21 ^d^	0.69	0.25	2.80	0.005
Post-implementation slope	0.24	0.06	3.95	<0.001

Note: ^a^ 0 = male, 1 = female; ^b^ 0 = non-Hispanic White, 1 = Hispanic or Latino, 2 = Asian American, 3 = other or multiple backgrounds; ^c^ 0 = participant was aged 18–20 at the time of assessment, 1 = participant was aged 21 or older at the time of assessment; ^d^ 0 = assessment occurred prior to Tobacco 21 implementation; 1 = assessment occurred after Tobacco 21 implementation.

## Data Availability

The dataset is available upon request from the second author at nmdoran@health.ucsd.edu.
